# Bovine Colostrum‐Derived Extracellular Vesicles Impair Cancer Cell Proliferation Through Transcriptional Dysregulation

**DOI:** 10.1002/jev2.70361

**Published:** 2026-09-01

**Authors:** Claudia Betsabé Huesa‐Carballo, Bruno Puga, Susana Bravo, Manuel Rodríguez‐Pérez, Yessica Domínguez‐Novoa, Julio Iglesias‐García, Brígida Hermida, Isolina Raña, María Teresa Antelo, Rafael López‐López, Miguel Abal, Jorge Barbazán

**Affiliations:** ^1^ Translational Oncology Laboratory (Oncomet) Health Research Institute of Santiago de Compostela Santiago de Compostela Spain; ^2^ University of Santiago de Compostela Santiago de Compostela Spain; ^3^ Proteomics Unit Health Research Institute of Santiago de Compostela Santiago de Compostela Spain; ^4^ ISCIII Centro de Investigación Biomédica en Red de Cáncer (CIBERONC) Madrid Spain; ^5^ Department of Gastroenterology and Hepatology Health Research Institute of Santiago de Compostela, University Hospital od Santiago de Compostela Santiago de Compostela Spain; ^6^ Xearte Brigitte Santiago de Compostela Spain; ^7^ Asociación de Empresarias Rurais ASER Cerceda Spain; ^8^ Granxa Devesa Langueirón Ponteceso Spain; ^9^ Medical Oncology Department University Clinical Hospital of Santiago de Compostela Santiago de Compostela Spain

**Keywords:** bovine colostrum, cell growth arrest, chromatin remodelling, extracellular vesicles, gastrointestinal cancer, proteomics, RNA, transcription

## Abstract

Milk‐derived extracellular vesicles (EVs) are a promising source of molecules with therapeutic potential. Bovine colostrum is particularly enriched in EVs, which carry cargo of proteins involved in immune regulation, development and cellular signalling. Some studies have explored their role as bioactive anti‐cancer agents, however, their mechanistic effects remain underexplored. Here, we show that colostrum‐derived EVs (Col‐EVs) exert anti‐proliferative effects in gastrointestinal cancer models, including cell lines and patient‐derived organoids, which is independent of apoptosis induction. Using a multi‐modal approach combining proteomics, imaging and functional assays, we demonstrate that Col‐EVs induce a reversible growth‐arrest state, characterized by widespread transcriptional and RNA‐processing dysregulation, chromatin compaction, nuclear reorganization and cytoskeletal remodelling. Proteomic analyses reveal that Col‐EV treatment disrupts key components of the transcriptional machinery and cell cycle regulatory pathways, effects that are reversible upon EV withdrawal and can be rescued pharmacologically using an EZH2 inhibitor. Col‐EVs enhance the sensitivity of cancer cells as well to DNA‐targeting chemotherapies such as 5‐fluorouracil, indicating their potential as modulatory adjuvants rather than cytotoxic agents. Overall, our findings reveal that Col‐EVs can reversibly suppress cancer cell proliferation by reprogramming transcriptional and nuclear architecture, offering a natural, biocompatible strategy for modulating tumour growth and sensitizing cancer cells to conventional therapies.

## 1 Introduction

Milk is a nutrient‐rich biological fluid that plays a central role in neonatal development, immune system maturation and gut homeostasis (Kalbermatter et al. 2021; Rodríguez‐Camejo et al. 2023; Walker and Iyengar 2015). Beyond its classical nutritional components, which include proteins, fats, carbohydrates, vitamins, and minerals, milk contains extracellular vesicles (EVs), nano‐sized lipid bilayer particles capable of transferring bioactive cargos such as proteins, RNAs, lipids, and metabolites between different cells, which don't have self‐replicative abilities (Barathan et al. 2024; Muttiah and Law 2025). These milk‐derived EVs (mEVs) have been implicated in a wide range of physiological processes, including modulation of gut barrier integrity (Tong et al. 2023; Aarts et al. 2021), immune responses (Rodríguez‐Camejo et al. 2023; Kim et al. 2025; Vahkal et al. 2024), and microbiota composition (Walker and Iyengar 2015; Muttiah and Law 2025; Du et al. 2021), thereby shaping health outcomes from infancy into adulthood.

Recent studies have demonstrated that mEVs are resistant to gastrointestinal degradation (Tong et al. 2023; Yung et al. 2024), enabling their oral bioavailability and functional uptake by gut epithelial cells and even systemic distribution to distal organs, including the liver and tumours. This stability, coupled with their biocompatibility and low immunogenicity, positions mEVs as promising candidates for oral nanotherapeutics and drug delivery vehicles, with applications ranging from inflammatory bowel disease and gut‐liver axis disorders to systemic modulation of immune responses and cellular signalling (Dai et al. 2024; Marsh et al. 2025; Amthaniwala et al. 2025). Interestingly, a recent study has also shown that bovine mEVs can reduce primary tumour burden in colorectal and breast cancer models by inducing tumour cell senescence (Samuel et al. 2021). However, while the potential of mEVs in supporting gut health and reducing inflammation is well recognized, their mechanistic effects as anti‐cancer agents deserve further exploration.

Bovine colostrum, the first milk produced post‐parturition, is particularly enriched in EVs containing high levels of immune‐modulatory proteins, antimicrobial peptides and growth‐regulatory factors (Muttiah and Law 2025; Santoro et al. 2023). These colostrum‐derived EVs (Col‐EVs) encapsulate a protein and RNA cargo that reflects the biological function of colostrum as a system supporting early immune training, epithelial barrier maturation and tissue development. Indeed, this specialized composition suggests that Col‐EVs may possess distinct biological activities compared to mature mEVs (Mecocci et al. 2024; Liu et al. 2024), with potential for differentially influencing cancer cell proliferation and survival. However, the mechanistic understanding of whether and how Col‐EVs could impact cancer cell proliferation remains limited.

In this study, we investigate the anti‐proliferative potential of bovine Col‐EVs on different gastrointestinal cancer models, including cancer cell lines, patient‐derived organoids and primary cells from the tumour microenvironment. We hypothesize that Col‐EVs induce a reversible growth arrest in cancer cells by interfering with transcriptional programs and promoting chromatin compaction, thereby suppressing proliferation without inducing apoptosis. Using a combination of proteomics, imaging, functional assays and pharmacological perturbations, we dissect the mechanistic pathways involved in this phenotype, providing insights into the therapeutic potential of Col‐EVs as natural, orally available bio‐nano modulators in cancer therapy.

## 2 Results

### 2.1 Comprehensive Characterization of Bovine Col‐EVs

**FIGURE 1:**
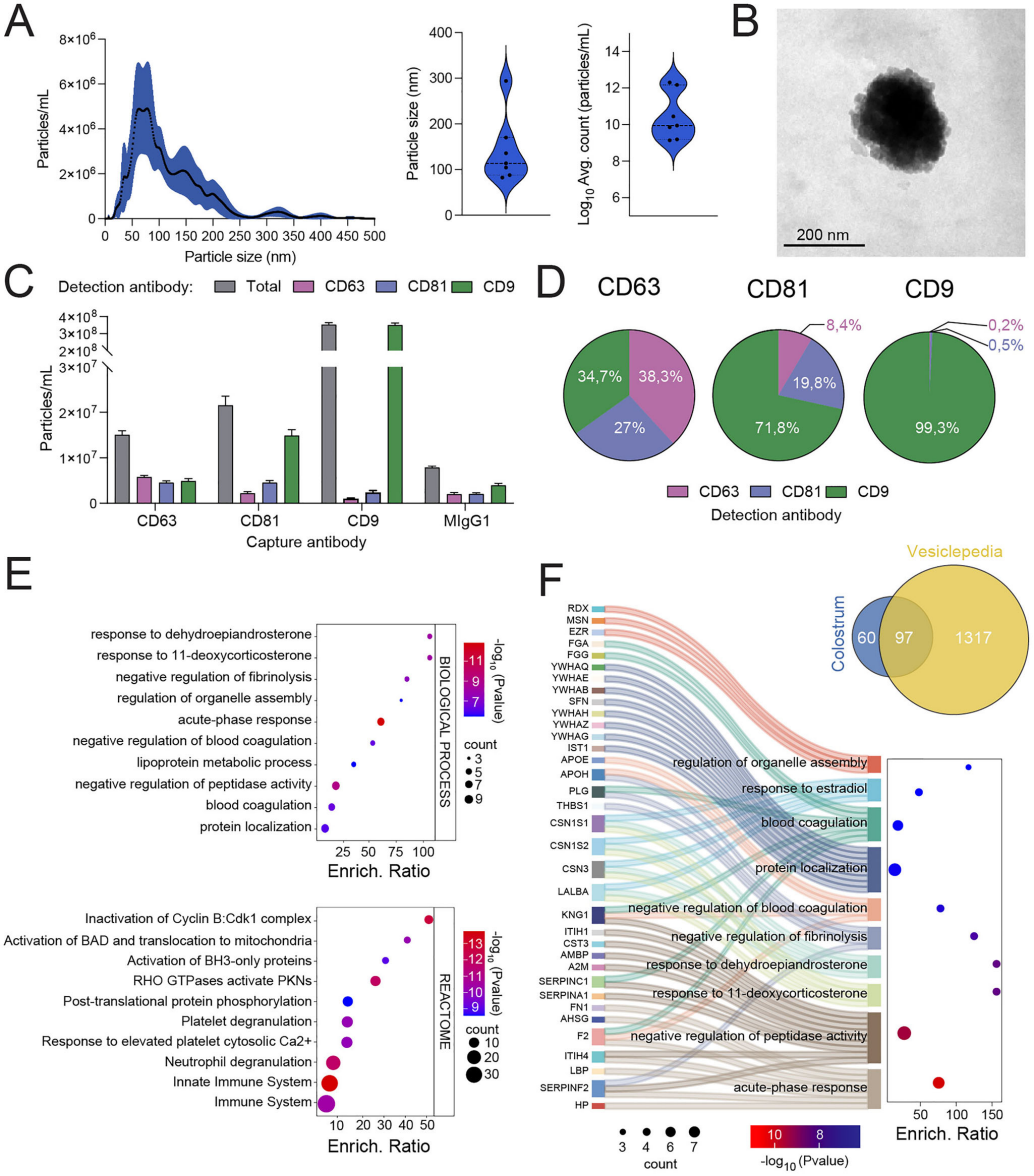
Characterization of bovine colostrum‐derived EVs. (A) Left panel: nanoparticle tracking analysis (NTA) showing the size distribution of two independent Col‐EV samples, with a primary peak around 100–150 nm (*N* = 8). Middle and right panels: violin plots of the quantification of particle size and concentration (*N* = 7), respectively. (B) Representative transmission electron microscopy (TEM) images confirming the spherical morphology and expected size range (100–200 nm) of isolated Col‐EVs. (C) Quantification of particle concentration using SP‐IRIS (*N* = 3). MIgG1 indicates the signal obtained using an isotype control. Error bars: SD. (D) Co‐expression analysis showing heterogeneity in tetraspanins profiles across the Col‐EV population (*N* = 3). (E): Gene Ontology (Biological Process) (upper panel) and Reactome pathway enrichment analyses (lower panel) of the Col‐EV DDA proteomic analysis. (F) Venn diagram and Sankey analysis demonstrating overlap with Vesiclepedia and highlighting functional categories of identified proteins. For EV proteomics samples were run in triplicates.

In order to understand the protein composition of EVs from bovine colostrum (Col‐EVs), we adapted an EV isolation protocol (Weiskirchen et al. 2023) based on sequential ultracentrifugation combined with different filtration steps (Figure S1). Nanoparticle tracking analysis (NTA) from two independent colostrum samples revealed a size distribution with a major peak around 100 nm and a long tail extending to 300 nm, consistent with the expected profile for exosome‐enriched EV populations (Figure 1A, left panel). This heterogeneity likely reflects both exosomal and small microvesicle populations. Quantification of replicate samples showed consistent particle concentration and size distribution (Figure 1A, centre and right). Moreover, Col‐EVs were visualized using transmission electron microscopy (TEM), confirming their vesicular morphology. Vesicles appeared spherical and sized in the 100–200 nm range (Figure 1B), supporting their classification as small EVs. Of note, other components such as lipid or casein aggregates were detected as part of the sample, as expected due to their high abundance in this sample type (Figure S2A). To further profile surface markers, we employed Single‐Particle Interferometric Reflectance Imaging Sensor (SP‐IRIS) analysis using antibody‐based capture and detection. Using capture antibodies against CD63, CD81 and CD9, we quantified the expression of canonical tetraspanins on individual vesicles (Figure 1C). CD9 was the most abundantly expressed tetraspanin, present on the majority of particles. CD81 and CD63 were detected at lower levels. As well, we found that CD63 and CD81 positive vesicles co‐expressed at different degrees other tetraspanins, while the vast majority of CD9‐isolated vesicles did not co‐expressed other markers (Figure 1D). These results validate the isolation methodology and indicate phenotypic heterogeneity within the isolated Col‐EV population.

To further characterize the proteome of Col‐EVs, we performed proteomic analyses using data‐depending acquisition (DDA) mass spectrometry. Using this approach, over 150 specific proteins were detected with less than 1% of FDR (false discovery rate). Further analyses showed an enrichment in pathways associated with inflammation, coagulation and complement activation, as revealed by both Gene Ontology (Biological Process) and Reactome pathway analyses (Figure 1E, Supporting Information Excel File). These results are in line with previous reports of colostrum proteomics, which consistently described colostrum as a fluid that contains high levels of antimicrobial peptides, immunoglobulins, complement proteins, and factors involved in wound healing and epithelial maturation (Samuel et al. 2021; Santoro et al. 2023; Mecocci et al. 2024) Notably, several proteins involved in organelle assembly and steroid responses were also enriched, indicating possible broader functional roles for these EVs. The detection of proteins related to organelle assembly and steroid responses suggests that Col‐EVs may also participate in intracellular signalling and metabolic priming, potentially supporting early tissue development and endocrine regulation. Comparison with the Vesiclepedia database (Chitti et al. 2023) revealed a substantial percentage of proteins associated with EVs (Figure 1F, Venn diagram and Supporting Information Excel File), reinforcing the presence of EVs in the sample. Such proteins included fibrinogen subunits (FGA, FGB, FGG), coagulation factors, and stress‐response proteins such as HP, APOE and LBP. Sankey visualization linked these proteins to functional categories such as regulation of organelle assembly, response to hormones (e.g., estradiol), and negative regulation of proteolytic activity (Figure 1F and Supporting Information Excel File).

Overall, these data indicate that bovine Col‐EVs possess a specialized protein cargo with potential immunomodulatory, structural and regulatory properties.

### 2.2 Col‐EVs Inhibit Gastrointestinal Cancer Cell Proliferation Without Inducing Cell Death

**FIGURE 2:**
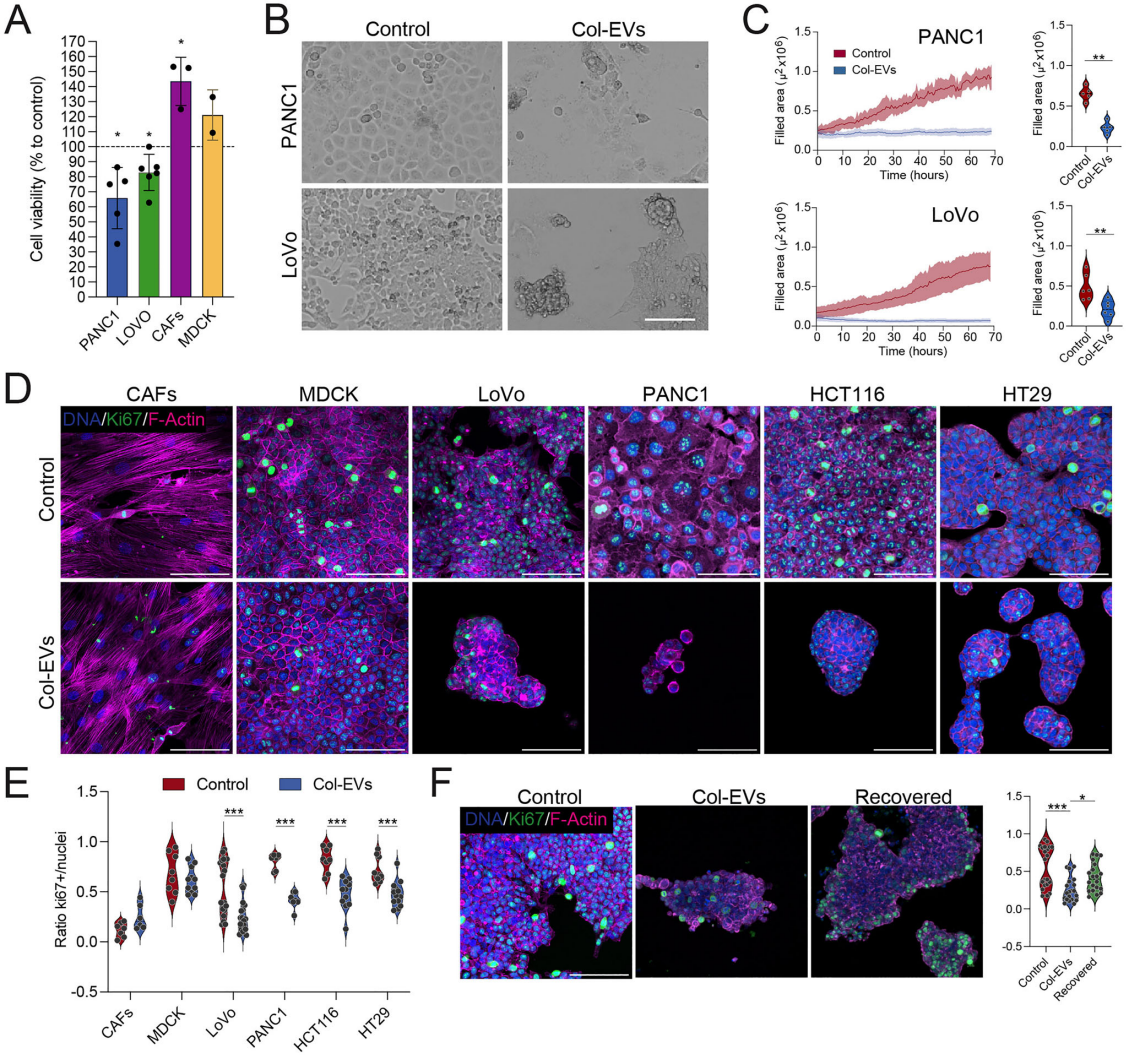
Col‐EVs impair cancer cell proliferation without inducing cell death. (A) Viability assays in colorectal (LoVo), pancreatic (PANC1) cancer cells, and in primary patient‐derived CAFs and MDCK cells upon Col‐EV treatment. Error bars: SD. One sample *t* test *p < 0.05 (*N* = 5 for PANC1 95% CI [−59.52 to −8.819]; *N* = 6 for LoVo 95% CI [−29.76 to −4.481]; *N* = 3 for CAFs 95% CI [3.580 to 83.55]; *N* = 2 for MDCK). (B) Representative time‐lapse images of PANC1 and LoVo cells 72 h after either control or Col‐EV treatment. (C) Left panels: quantification of PANC1 and LoVo filled area over time from videomicroscopy experiments. Scale bar: 100 µm. Right panels: violin plots of the quantification of total filled area at *t* = 72 h post‐treatment. Data from at least *n* = 8 independent positions for *N* = 5 (PANC1) and *N* = 6 (LoVo) experiments. Mann–Whitney Nonparametric test **p < 0.01; LoVo CI95.89% [−0.4880 to −0.07806]; PANC1 CI96.83% [−0.5415 to −0.2925]. (D) Confocal images of control and Col‐EV treated cell lines. Scale bar: 100 µm. (E) Quantification of Ki67 positive cells per total count of DAPI positive cells. ***p < 0.001; Kruskal–Wallis ANOVA test. Data from at least 15 images from at least *N* = 2 independent experiments. (F) Left panel: representative images of Ki67‐stained (green) control, Col‐EV treated and recovered LoVo cells. F‐actin (phalloidin): magenta. DNA (DAPI): blue. Scale bar: 50 µm. Right panel: quantification of Ki67 positive cells per total count of DAPI positive cells. *p < 0.05; ***p < 0.001; Kruskal–Wallis ANOVA test/Dunn's multiple comparisons. Data from *n* = 20 images from *N* = 2 independent experiments.

To determine the potential biological effects of Col‐EVs on cancer cells, we treated a panel of gastrointestinal epithelial cancer cell lines, primary cancer‐associated fibroblasts (CAFs), and a non‐transformed epithelial cell line (MDCK) with Col‐EVs. Alamar Blue viability assays performed 72 h post‐treatment revealed a significant decrease in cell number/metabolic activity in colorectal (LoVo) and pancreatic (PANC1) cancer cells, while MDCKs remained unaffected and CAFs even increased their proliferation rates (Figure 2A), suggesting a potential selective targeting of epithelial cancer cells without elevated toxicity in normal cells. As well, to assess the impact of EVs on cell growth dynamics, we performed time‐lapse microscopy assays over a period of 72 h. Col‐EV‐treated PANC1 and LoVo cells exhibited a marked inhibition of proliferation, as they failed to expand into a monolayer during exploratory experiments using time‐lapse imaging (Figure 2B and Supporting Information: Video 1). Quantitative analysis of the cancer cell area filled over time confirmed an important reduction in growth compared to controls (Figure 2C). In addition to a reduction in proliferation, we observed that Col‐EVs induced a distinct morphological phenotype, forming compact, spheroid‐like aggregates which were particularly evident in LoVo cells (Figure 2B, Figure S3A), suggesting a decreased cell‐substrate adhesion and increased cell‐cell compaction. Interestingly, this phenotype was reproduced in an extended panel of colorectal cancer cell lines (HT29 and HCT116), while CAFs and MDCKs remained unaffected (Figure 2D). This phenotype is consistent with a dewetting‐like response and could reflect the loss of adhesion and polarity associated with cancer cell‐specific EV‐induced growth arrest.

To determine whether the observed effects were due solely to growth arrest or also involved cell death, we performed cell death staining, which revealed no significant differences between control and Col‐EV treated cells (Figure S3B), indicating that the treatment did not induce apoptosis in two selected cancer cell lines. Contrarily, Ki67 immunostaining revealed a substantial reduction in the number of actively cycling cells under Col‐EV treatment, but, importantly, only in transformed cancer cells (Figure 2D,E). As well, we observed that, when EVs were removed from the culture medium, Ki67 positivity and monolayer organization were largely restored (Figure 2F, right panel), demonstrating that the anti‐proliferative effects of Col‐EVs are reversible.

**FIGURE 3:**
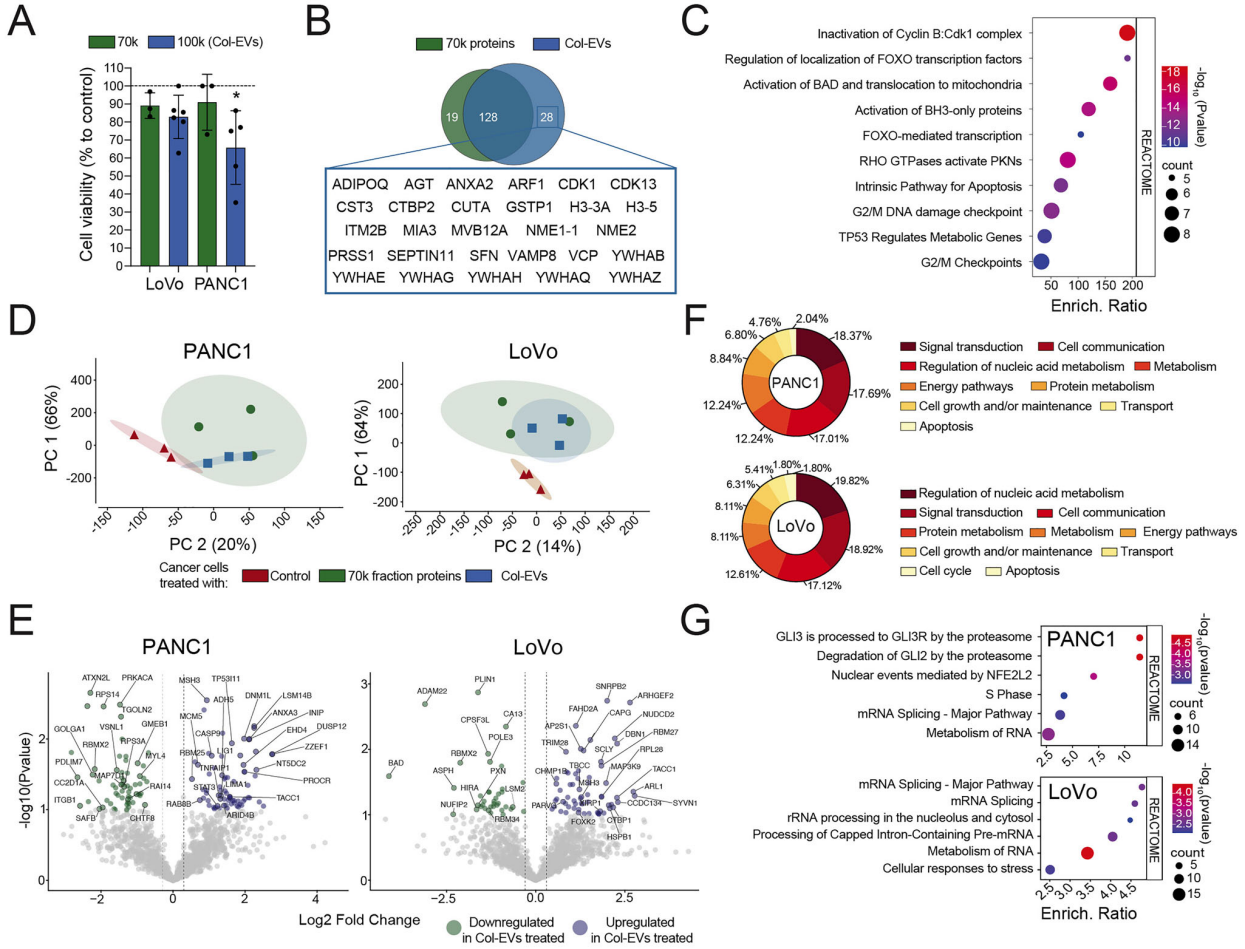
Col‐EVs‐specific proteins induce growth arrest in cancer cells through a mechanism compatible with transcriptional repression. (A) Viability assays comparing the effects of 70k (protein‐rich) and 100k (Col‐EV‐enriched) fractions on LoVo and PANC1 cells. Error bars: SD. One sample *t* test *p < 0.05; LoVo 95% CI [−28.47 to 6.755]; PANC1 95% CI [−59.52 to −8.819]. Data show at least three independent experiments per condition (B) Venn diagram displaying unique proteins identified in the Col‐EV fraction. (C) Reactome pathway enrichment of Col‐EV exclusive proteins showing involvement in cell cycle regulation, FOXO‐mediated transcription, and G2/M checkpoint control. For EV proteomics samples were run in triplicates. (D) SWATH‐MS proteomics principal component analysis (PCA) of control, 70k and Col‐EV treated PANC1 and LoVo cells. Data obtained from three independent experiments per condition. (E) Volcano plots showing widespread dysregulation of proteins in PANC1 and LoVo cells upon treatment with Col‐EVs. Fold change levels were calculated using 70k‐treated cells as controls. Thresholds: *P* value: 0.01; Fold Change: 1.3/−1.3. Data obtained from three independent experiments. (F, G) Functional enrichment analyses (F:Funrich; G: Reactome) highlighting the top 6 dysregulated pathways in PANC1 and LoVo cells upon Col‐EV treatment, compared with the treatment with the 70k protein fraction. Data obtained from three independent experiments.

In order to exclude that the observed phenotype could be an artifact of the EV isolation method, we optimized an alternative protocol in which colostrum samples were precleared from caseins and other non‐EV components using size exclusion chromatography (SEC) (Figure S4A,B). Although the average particle size was similar in samples processed in parallel using SEC and ultracentrifugation, EV concentration was significantly reduced upon SEC, as expected when introducing an additional purification step (Figure S4C). Still, cancer cells treated with SEC‐isolated Col‐EVs rendered a similar phenotype than ultracentrifugation (Figure S4D,E). This was further reinforced by cell viability assays comparing Col‐EVs isolated in the 100,000 × *g* (100k) fraction with material recovered in the preceding 70,000 × *g* (70k) intermediate pellet from the same sample. While both fractions contain comparable numbers of total particles (Figure S2C), canonical CD63+ and CD81+ EV populations were reduced in the 70k fraction, suggesting that the two fractions represent compositionally distinct particle populations (Figure S2B–E). We observed that only the 100k fraction affected cancer cell proliferation (Figure 3A), indicating that the anti‐proliferative activity is specific to the Col‐EV fraction of the colostrum. Finally, and to verify that such effects were specifically colostrum‐associated, we treated cancer cells with EVs isolated from matched samples of colostrum (Col‐EVs) and mature milk (Milk‐EVs), which ranged in the same size and concentration values as SEC‐isolated Col‐EVs (Figure S5A). We found that Milk‐EVs exerted an intermediate phenotype compared to Col‐EVs, both at the general and proliferative phenotypes (Figure S5B,C), suggesting that specific components of colostrum‐derived EVs were, at least, partially responsible for the observed cellular effects.

Overall, these results indicate that colostrum‐derived EVs could suppress the proliferation of epithelial cancer cells without inducing cell death, instead promoting a reversible growth‐arrested state. This is accompanied by striking changes in cellular architecture and adhesion, suggesting that Col‐EVs interfere with both proliferative and mechanical cues in cancer cells.

### 2.3 The Anti‐Proliferative Effects of Col‐EVs Are Linked to Transcriptional Dysregulation Programs

The observation that biological activity was restricted to the 100k Col‐EV‐enriched fraction suggested that specific molecular components associated with Col‐EVs are responsible for their anti‐proliferative phenotype. We therefore hypothesized that functionally relevant proteins are selectively enriched in this fraction. To test this hypothesis and to explore potential regulatory pathways underlying growth suppression, we performed comparative proteomic profiling of the 100k and 70k fractions, which revealed a total of 28 proteins unique to the 100k Col‐EV sample. These proteins include nuclear proteins such as histones H3‐3A and H3‐5, transcriptional regulators CDK1 and CDK13, and annexins, among others (Figure 3B and Supporting Information Excel File). Pathway enrichment analyses revealed cell cycle regulation, FOXO‐mediated transcription, and G2/M checkpoints as some of the main functions in which proteins specific to Col‐EVs were found to be involved, which could be linked to the observed effects over cell proliferation (Figure 3C and Supporting Information Excel File).

To better understand the specific effects of Col‐EVs on cancer cells, we performed quantitative whole‐cell SWATH‐MS proteomics on LoVo and PANC1 cells treated with either the 70k fraction or Col‐EVs. Principal component analysis (PCA) showed that, as expected, the major differences were observed between control cells and those treated with both fractions (Fig 3D, Figure S5D and Supporting Information Excel File). However, samples treated with the 70k fraction and Col‐EVs also clustered separately from each other in both cell lines (Figure 3D), suggesting that Col‐EVs could exert a distinct and specific effect on cancer cells beyond that of the protein‐rich 70k pellet. Volcano plot analyses revealed that treatment with Col‐EVs led to a widespread dysregulation of protein expression across LoVo and PANC1 cells (Figure 3E and Supporting Information Excel File). Col‐EV treatment altered the abundance of key regulators of gene expression and cellular architecture. This included downregulation of RNA splicing factors (e.g., RBM25, SNRPB2), chromatin‐associated enzymes (e.g., DNMT1, PRMT5), and structural proteins involved in mitotic progression (e.g., TACC1). In parallel, we observed dysregulation of proteins involved in cytoskeletal dynamics and adhesion, including CAPG, a modulator of actin filament capping; MYH9, a non‐muscle myosin heavy chain important for cell contractility; and integrin β1 (ITGB1), a major mediator of cell‐ECM interactions, consistent with the observed cellular phenotypes.

To further explore the biological relevance of these proteomic shifts, we performed pathway enrichment analysis using FunRich and Reactome. In both LoVo and PANC1 cells, treatment with Col‐EVs led to a significant enrichment of dysregulated proteins involved in transcriptional regulation, RNA metabolism and cell cycle progression (Figure 3F and Supporting Information Excel File). Notably, Reactome analysis highlighted the disruption of pathways such as mRNA splicing, S phase progression, and the processing of capped pre‐mRNAs (Figure 3G and Supporting Information Excel File), processes that are relevant for the transcriptional and post‐transcriptional control of gene expression.

Collectively, these results suggest that Col‐EVs mediate their anti‐proliferative effects by targeting core transcriptional and RNA processing pathways, thereby depriving cancer cells of the molecular machinery required to sustain cell cycle progression.

### 2.4 Col‐EVs Induce Reversible Chromatin Condensation and Nuclear Reorganization

**FIGURE 4:**
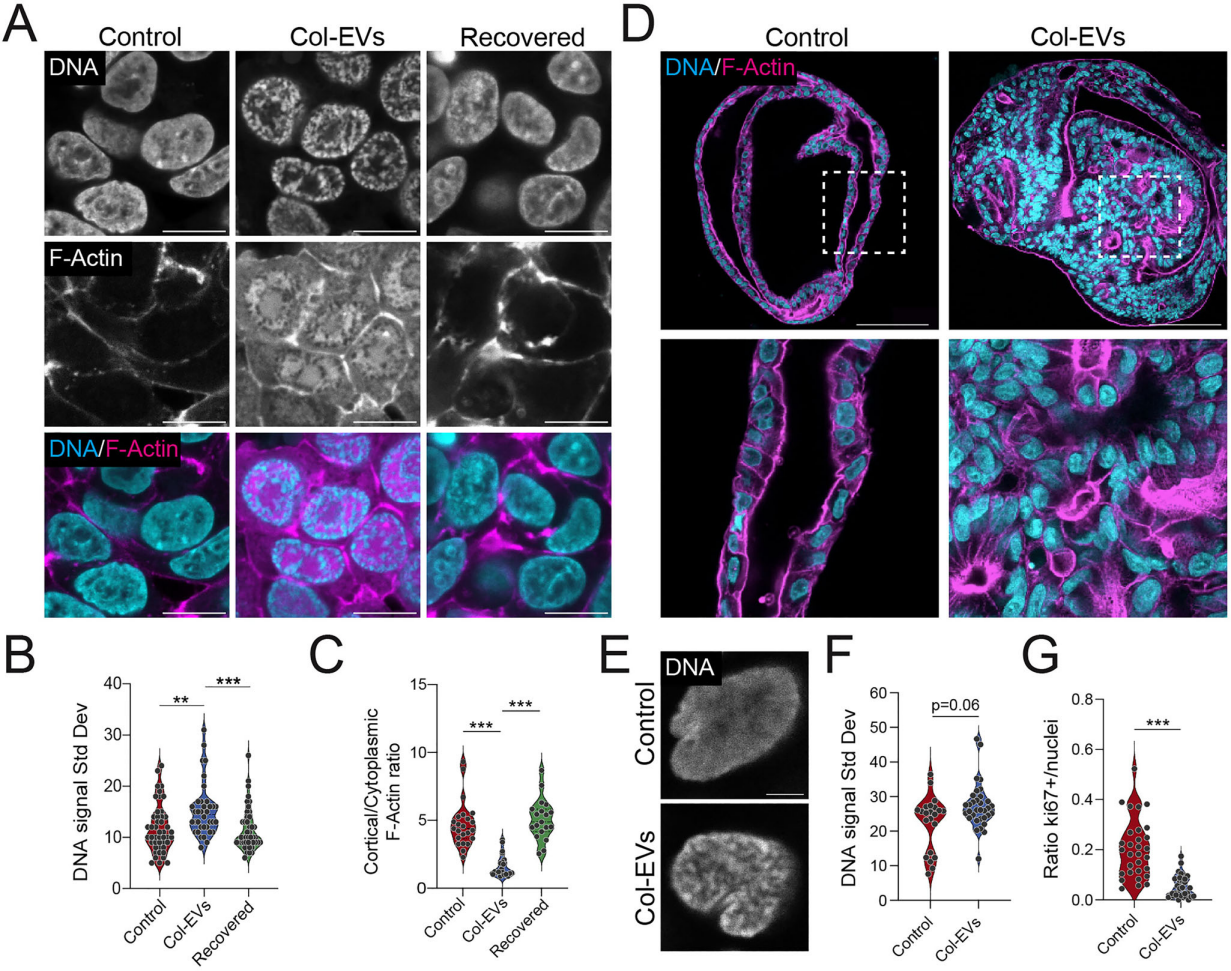
Col‐EVs induce reversible chromatin compaction and nuclear remodelling. (A) Representative confocal images of DAPI‐stained nuclei from LoVo cells, control, Col‐EV treated or recovered. Scale bar: 5 µm (B) Quantification of the standard deviation of the nuclear signal/nucleus, confirming reversible chromatin compaction upon EV removal. Data represent at least 35 analysed nuclei from *n* = 2 independent experiments. **p < 0.01, ***p < 0.001. (C) Quantification of F‐Actin cortical/cytoplasmic ratios for at least 20 analysed nuclei from *n* = 2 independent experiments. ***p < 0.001. (D) Representative confocal images of DAPI and F‐Actin stained patient‐derived PDAC organoid models, control and Col‐EV‐treated. Scale bar: 100 µm. Lower panels, insets of selected organoid regions (E) Confocal images of DAPI‐stained nuclei from PDAC organoid models, control and Col‐EV‐treated (F) Quantification of the standard deviation of the nuclear signal/nucleus. Data represent at least 22 analysed nuclei from *N* = 3 independent experiments. (G) Quantification of Ki67 positive cells per total count of DAPI positive cells. ***p < 0.001. Data from at least 30 images from *N* = 3 independent experiments. Statistical analysis for Panels B, C, F and G was performed using the Kruskal–Wallis test followed by Dunn's multiple comparisons test.

Given that proteomic analyses revealed an enrichment of proteins involved in transcriptional regulation, RNA processing and cell cycle control, we hypothesized that the observed growth arrest phenotype may be associated with structural changes at the level of nuclear architecture. To check this, we examined the nuclear morphology and chromatin organization using high‐resolution confocal microscopy. Upon treatment with Col‐EVs, LoVo cells displayed alterations in their nuclear architecture, including visible DAPI‐bright foci (Figure 4A,B). These changes were largely absent in control cells, in which interphase nuclei showed smooth and homogeneous DNA distribution. Although at different levels, the same effect was observed in some of the other cancer cell lines treated both with Col‐EVs coming from ultracentrifugation (Figure S3C,D) and SEC (Figure S4F,G), while non‐transformed CAFs and MDCK remained unaffected (Figure 3C,D). Finally, only a mild effect could be detected in cells treated with Milk‐EVs (Figure S5E,F), suggesting that this phenotype could be Col‐EV specific.

Interestingly, the few Col‐EV treated cells able to enter mitosis showed an aberrant chromosomal hypercompaction phenotype (Figure S6A–C), which did not prevented cells from undergoing M‐phase, but likely resulting in aberrant incomplete division that could be leading to proliferation arrest. Indeed, Ki67, which normally locates around individual chromosomes acting as a surfactant to prevent chromosome clumping and aberrant division (Cuylen et al. 2016), was found to be located around the main metaphasic chromosome mass (Figure S6A,D), likely unable to properly localize due to chromatin compaction. All these changes were accompanied by a reorganization of F‐actin pools, not able to localize to the cell cortex during interphase/division and remaining mislocalized over the entire cytoplasm in Col‐EV treated cells (Figure 4A,C; Figure S6A). Concordantly with the previously observed Ki67+ ratios (Figure 2F), nuclear DNA distribution (Figure 4A,B), Ki67 localization (Figure S6A,D) and F‐Actin pools (Figure 4A,C) returned to normal levels upon Col‐EV withdrawal, reinforcing the transitory/reversible effect of Col‐EVs.

It has been recently reported that osmotic stress affects nuclear organization and chromatin structure (McCreery et al. 2025). To exclude the possibility that the addition of Col‐EVs to the culture medium could change its osmolarity and thus generate artifacts, we evaluated the osmolarity of all experimental conditions. Increasing concentrations of D‐sucrose were used as positive controls to induce defined hyperosmotic conditions, which resulted in dose‐dependent chromatin compaction (Figure S7A,B). In contrast, osmolarity measurements revealed that supplementation with Col‐EVs, Milk‐EVs, SEC‐EVs, or control fractions did not significantly alter medium osmolarity compared with standard culture conditions (Figure S7C), discarding a direct effect of osmotic pressure over the observed phenotype.

To further validate the physiological relevance of this phenotype, we next examined the effects of Col‐EVs in patient‐derived PDAC organoid models. Unlike traditional 2D cultures, these 3D models more accurately recapitulate the complex architecture and biomechanical properties of the primary tumour. Importantly, organoids maintain the inherent apicobasal polarization and multicellular interactions characteristic of a glandular epithelium, providing a more robust system for studying structural disruption. Consistent with our observations in cell lines, we observed significant alterations in epithelial architecture and cytoskeletal organization, with organoids losing epithelial polarity (Figure 4D), as well as the same chromatin condensation phenotype previously reported (Figure 4E,F), which was accompanied by a significant reduction in proliferation (Figure 4G).

Together, these results show that the transcriptional dysregulation and growth arrest induced by Col‐EVs could be associated to reversible changes in chromatin condensation independent of osmotic stress.

### 2.5 Pharmacological Inhibition of Chromatin Compaction Reverses Col‐EV‐Induced Growth Arrest

**FIGURE 5:**
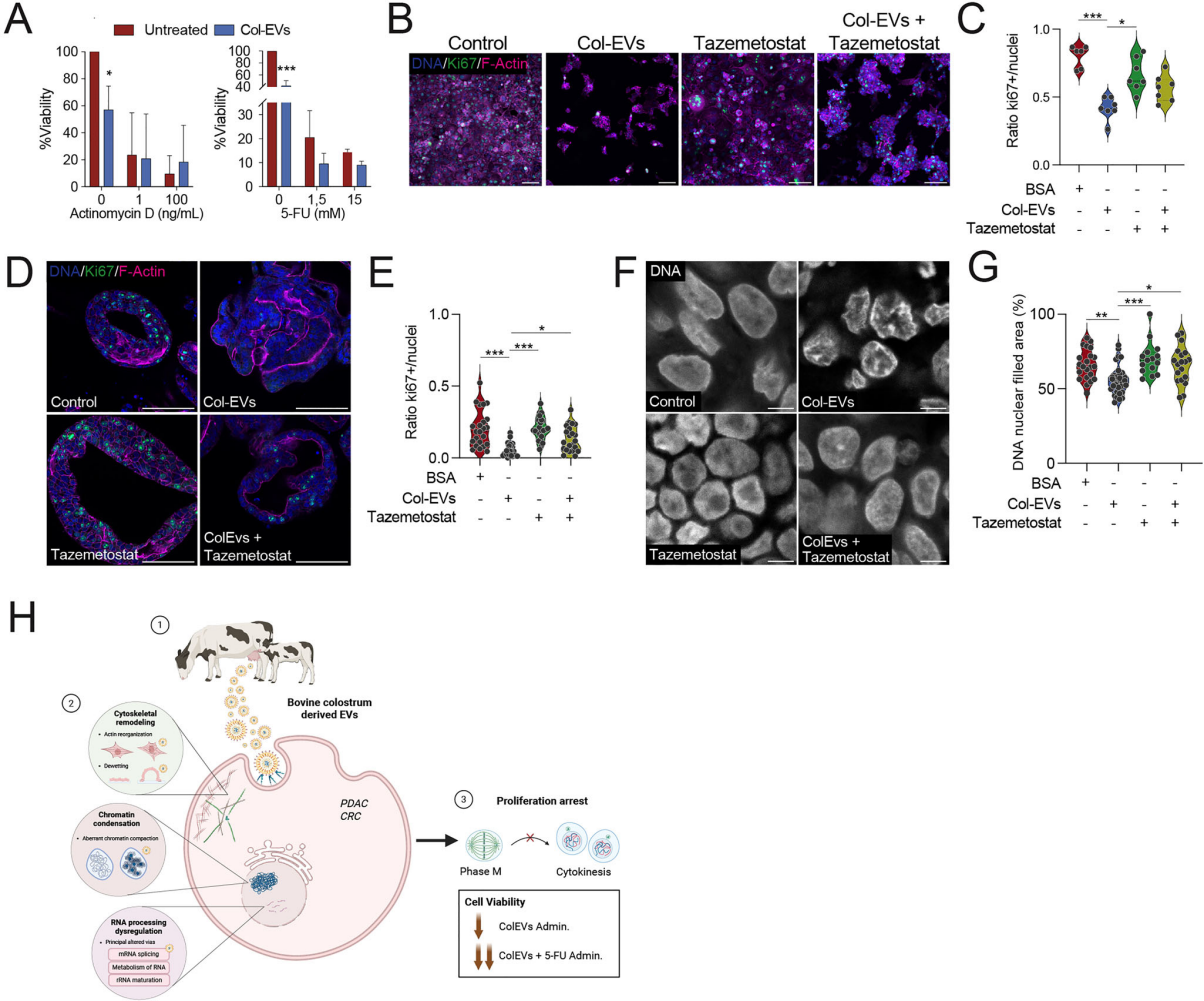
Pharmacological rescue of Col‐EVs‐induced effects. (A) Viability assays in PANC1 cells treated with increased doses of actinomycin D (left panel) or 5‐FU (right panel), with or without Col‐EVs (*n* = at least four independent replicates). ANOVA, Sidak's multiple comparisons test. Error bars: SD. *p < 0.05; ***p < 0.001; Actinomycin 95% CI [0.4807, 85.43]; 5‐FU 95% CI [43.04 to 72.86]. (B) Representative images of PANC‐1 cells treated with Col‐EVs and rescued by co‐treatment with tazemetostat (40 µM). Scale bar: 100 µm (C) Quantification of Ki67 positive cells per total count of DAPI positive cells. *p < 0.05; ***p < 0.001; ANOVA Kruskal–Wallis test/Dunn's multiple comparisons. Data from at least six images from *N* = 2 independent experiments. (D) Confocal images of PDAC organoid models showing rescue of the Col‐EVs‐phenotype upon treatment with tazemetostat (40 µM). Scale bar: 100 µm (E) Quantification of Ki67 positive cells per total count of DAPI positive cells. *p < 0.05; ***p < 0.001; ANOVA Kruskal–Wallis test/Dunn's multiple comparisons. Data from at least 22 images from *N* = 2 independent experiments. (F) Representative confocal images of DAPI‐stained nuclei from PDAC organoid models, controls, Col‐EV treated or rescued with tazemetostat 40 µM. Scale bar: 5 µm (G) Quantification of the % nuclear area covered by DNA in PDAC organoid models. Data represent at least 15 analysed nuclei from *N* = 2 independent experiments. *p < 0.05; **p < 0.01; ***p < 0.001; ANOVA Kruskal–Wallis test/Dunn's multiple comparisons. (H) Schematic representation of the proposed mechanism.

To further dissect the molecular mechanisms underlying Col‐EV effects, we first assessed whether the phenotype induced by Col‐EVs was primarily driven by transcriptional dysregulation. For this, we treated cells with actinomycin D, a potent inhibitor of RNA synthesis, alone or in combination with Col‐EVs, observing no synergistic effects in cancer cell viability upon combination of both treatments (Figure 5A, left panel). In contrast, when cells were co‐treated with Col‐EVs and 5‐fluorouracil (5‐FU), a chemotherapeutic agent whose primary mechanism of action involves inhibition of thymidylate synthase and incorporation into DNA, we observed an enhancement of cytotoxicity compared to 5‐FU alone (Figure 5A, right panel). These results, although indirect, suggest that Col‐EVs response could depend more on RNA. Notably, the anti‐proliferative effect of Col‐EVs alone was less pronounced than that of actinomycin D, supporting the idea that Col‐EVs exert a milder, more modulatory effect on transcription, possibly accounting for the reversibility of the phenotype observed upon EV removal.

Considering our proteomic data and the chromatin condensation effects observed in Col‐EV‐treated cells, we then hypothesized that preventing or relaxing chromatin condensation could restore nuclear accessibility and transcriptional activity. To test this, we pharmacologically modulated chromatin compaction using tazemetostat, a clinically approved EZH2 inhibitor that modulates chromatin structure by inhibiting H3K27 trimethylation, a repressive histone mark associated with chromatin compaction and gene silencing. Consistently, tazemetostat treatment partially restored Col‐EVs induced phenotype at the cell adhesion/polarity and Ki67 positivity levels, both in cancer cell lines (Figure 5B,C) and in PDAC organoids (Figure 5D,E; Figure S8), in which results were particularly significant, and accompanied by a rescue in Col‐EV‐induced chromatin condensation (Figure 5F,G).

Together, these findings support a model in which Col‐EVs induce growth arrest through chromatin compaction and transcriptional dysregulation, and demonstrate that this phenotype remains reversible through pharmacological targeting of chromatin epigenetic regulators.

## 3 Discussion

Our study demonstrates that bovine colostrum‐derived extracellular vesicles (Col‐EVs) exert an anti‐proliferative effect on gastrointestinal cancer cells, mediated through chromatin remodelling and transcriptional dysregulation. These effects are specific to epithelial tumour cells, reversible upon EV withdrawal, and not associated with classical apoptosis or overt cytotoxicity. This potentially selective and transient inhibition of proliferation suggests a regulatory, rather than destructive, interaction between Col‐EVs and recipient cancer cells.

The phenotypic changes observed both in cancer cell lines and in patient‐derived PDAC organoids, including reduced proliferation, chromatin compaction, and cytoskeletal reorganization, point to a global reprogramming of cellular architecture and gene expression. Proteomic analysis confirmed that Col‐EV treatment leads to the dysregulation of RNA processing and transcription‐related proteins, many of which are important for cell cycle progression and metabolic maintenance. Pathway enrichment further indicated an involvement of transcriptional and epigenetic regulatory networks, consistent with the observed chromatin condensation and reversible growth arrest. Although the global response to Col‐EVs was conserved across both LoVo and PANC1 cells, some differences were observed in the specific pathways dysregulated in each model. These differences likely reflect intrinsic characteristics of the two tumour types, including distinct metabolic requirements, signalling dependencies and baseline transcriptional states. Such context‐dependent responses may influence how individual cell types process and respond to Col‐EV cargo while still converging toward a common phenotype characterized by transcriptional dysregulation and growth arrest. In addition, differential activation of stress‐response pathways, including NFE2L2‐associated networks, may contribute to the cell‐line‐specific proteomic signatures observed following Col‐EV treatment and warrants further investigation.

These results, together with the pharmacological rescue experiments using the EZH2 inhibitor tazemetostat, these findings suggest that Col‐EVs could be exerting their effects through the modulation of chromatin accessibility and transcriptional activity. The rescue of the phenotype and its sensitivity to chromatin‐modifying drugs support a model in which Col‐EVs alter endogenous chromatin remodelling and gene regulatory programs in recipient cells.

One important feature of the Col‐EV‐induced phenotype is its reversibility. Upon removal of EVs from the culture medium, cancer cells partially restored normal morphology, Ki67 expression and proliferation as well as DNA architecture. This reversible quiescent‐like state bears resemblance to tumour dormancy or transient growth arrest, phenomena with therapeutic implications for controlling residual disease and preventing relapse, which should be carefully considered when designing future combinatory experimental therapeutic regimens. However, the capacity to induce a pause in proliferation without causing cell death could be particularly beneficial in reducing tumour burden while sparing surrounding tissues from cytotoxic damage. Additionally, we demonstrate that pharmacological inhibition of transcription using actinomycin D phenocopies the effects of Col‐EVs, while co‐treatment with 5‐fluorouracil (5‐FU) reveals heightened sensitivity in EV‐treated cells. These findings suggest that Col‐EVs may sensitize cancer cells to DNA‐damaging or anti‐metabolic drugs by interfering with transcriptional and chromatin regulatory programs. From a mechanistic standpoint, this convergence on transcriptional dysregulation underscores a possible vulnerability in cancer cells that could be exploited therapeutically in the future.

The structural changes induced by Col‐EVs extend beyond the nucleus. We observed a significant disorganization of the actin cytoskeleton, which impaired epithelial polarity, particularly in cancer organoids. These changes may be altering factors that are important for metastasis and invasion. Although our study did not directly assess migration or invasion, the morphological shift toward a less adherent, more compact phenotype suggests potential effects on epithelial‐to‐mesenchymal transition (EMT) and metastatic competence. Interestingly, previous reports have shown that, while mEVs prevent primary tumour growth, they could be linked to an acceleration of tumour progression and metastasis (Amthaniwala et al. 2025). In light of our findings when comparing mature milk EVs with Col‐EVs effects over cancer cells, it is reasonable to hypothesize that invasion/migration could as well differ, guaranteeing further exploration.

From a translational perspective, the preferential activity of Col‐EVs toward transformed epithelial cells, while sparing non‐transformed epithelial cells and fibroblasts, suggests a potentially favourable therapeutic window. Although the mechanisms underlying this selectivity were not directly investigated in the present study, several observations provide plausible explanations. For instance, cancer cells are characterized by elevated proliferative demands, increased transcriptional output, altered chromatin dynamics, and a greater dependence on RNA‐processing pathways to sustain continuous growth (Hanahan 2022; Sengupta and George 2017). In contrast, normal epithelial cells, which maintain lower proliferative rates and more stable transcriptional programs, may better tolerate regulatory changes induced by Col‐EVs. Additional factors, including differences in EV uptake efficiency, metabolic state, and cellular stress‐response pathways, may also contribute to the differential sensitivity observed between normal and transformed cells. While these possibilities remain to be investigated, they represent important avenues for future mechanistic studies.

An additional consideration when interpreting our findings is the cross‐species nature of the experimental system. Although Col‐EVs originate from bovine colostrum, many of the cellular pathways affected in our study, including transcriptional regulation, RNA processing, and chromatin organization, represent core regulatory programs that are highly conserved across species and particularly in mammals (Hübner et al. 2013; Will and Lührmann 2011; Bylino et al. 2020). While some post‐transcriptional regulatory mechanisms might display species‐specific features (Schaefke et al. 2018), the core transcriptome across different tissues displays functional conservation between cattle and humans (Yao et al. 2022). Therefore, it is biologically plausible that regulatory molecules carried by bovine Col‐EVs can modulate equivalent cellular networks in human cells. Importantly, our findings are supported by a growing body of literature demonstrating that bovine milk‐ and colostrum‐derived bioactive components can exert biological effects across species boundaries (Du et al. 2021; Samuel et al. 2021; Sanwlani et al. 2020; Prasadani et al. 2024; Ross et al. 2021). Together, these observations support the concept that bovine‐derived EVs can mediate biologically relevant cross‐species signalling and provide a plausible framework for the effects observed in this study.

Col‐EVs could offer several advantages. They are naturally occurring, biocompatible, and derived from a widely consumed food product. Their ability to survive gastrointestinal transit and exert systemic effects supports the feasibility of oral or local delivery. Furthermore, the targeting of epithelial tumour cells without much impact in patient‐derived fibroblasts or normal epithelial cells, suggests a possible favourable safety profile. The identification of distinct EV cargo and downstream proteomic signatures also provides opportunities for biomarker discovery.

Future studies should focus on in vivo validation of these findings, including biodistribution, therapeutic efficacy, and safety in immunocompetent models of gastrointestinal cancer. It will also be critical to define the molecular mediators within the EV cargo responsible for transcriptional dysregulation and chromatin remodelling. Targeted engineering or enrichment of these components could enhance the therapeutic utility of Col‐EVs and lead to next‐generation EV‐based biotherapeutics.

In summary, our findings uncover a novel mechanism by which bovine colostrum‐derived EVs modulate cancer cell biology. By inducing a reversible, transcriptionally altered state characterized by chromatin condensation and cytoskeletal remodelling, Col‐EVs could represent a promising, naturally derived strategy for controlling tumour proliferation. We believe that this work expands the functional landscape of dietary EVs and provides initial evidence for their possible future development as therapeutic agents in oncology.

## 4 Materials and Methods

### 4.1 Colostrum and Milk Sample Collection and Processing

Bovine colostrum samples were obtained from two healthy Holstein dairy cows during the immediate postpartum period from granxa Devesa Langueirón (Ponteceso, Galicia, Spain). Animals were maintained under standard husbandry conditions and milked according to routine farm management practices. Colostrum was collected within the first 12 h after parturition using a mechanical milking system under aseptic conditions. Prior to collection, teats were thoroughly cleaned and disinfected to minimize microbial contamination. The first streams of secretion were discarded, and colostrum was subsequently collected into clean containers. Immediately after collection, colostrum samples were filtered through sterile gauze to remove coarse debris and particulate material and immediately frozen at −20°C until further use. All samples underwent a single freeze‐thaw cycle prior to EV isolation.

For matched comparative analyses, a parallel mature milk sample was collected from one animal, 15 days after parturition, corresponding to the transition from colostrum to established lactation. Milk collection was performed using the same hygienic procedures and mechanical milking system as described for colostrum. Mature milk samples were subjected to identical filtration, processing, aliquoting, and storage conditions to ensure experimental consistency. This paired sampling strategy enabled direct comparison of EVs derived from early colostrum and mature milk under controlled biological and technical conditions.

### 4.2 Isolation of Colostrum‐Derived Extracellular Vesicles (Col‐EVs)

For Col‐EVs isolation, first, bovine colostrum (40 mL) was diluted 1:1 with sterile phosphate‐buffered saline (PBS) and centrifuged at 3000 × *g* for 15 min at 4°C to remove fat and cellular debris. The upper fat layer was carefully removed, and the pellet was discarded. The resulting supernatant was transferred to ULTRACLEAR ultracentrifuge tubes (Beckman Coulter) and subjected to sequential differential ultracentrifugation using a 32SWT swinging bucket rotor (Beckman Coulter) as follows:

- 12,000 × *g*, 1 h, 4°C (discard fat layer)
- 30,000 × *g*, 1 h, 4°C
- 70,000 × *g* (first spin), 1 h, 4°C
- 70,000 × *g* (second spin), 1 h, 4°C

The final supernatant was filtered through 0.45 µm and 0.22 µm pore size syringe filters (Millipore). A final ultracentrifugation step was then performed at 100,000 × *g* for 2 h at 4°C. The resulting was resuspended in 2.5 mL of sterile, serum‐free DMEM/F12 medium, filtered through a 0.22 µm syringe filter, and stored at –80°C until further use.

The same protocol was applied to mature milk samples in order to isolate Milk‐EVs. This protocol yielded similar particle concentrations to previous works (Tong et al. 2023; Vahkal et al. 2024; Samuel et al. 2021).

### 4.3 Isolation of Col‐EVs Using Size Exclusion Chromatography (SEC)

Bovine colostrum samples were processed following the ultracentrifugation‐based isolation protocol described above, with the inclusion of an additional EDTA pretreatment step. After the initial pre‐clearing centrifugation at 3000 × *g* for 15 min at 4°C, the resulting supernatant was treated with 0.25 µM EDTA (1:1, v/v) to reduce the presence of casein micelles. The samples were then subjected to the same sequential differential ultracentrifugation steps as described for Col‐EV isolation, with a final ultracentrifugation at 100,000 × *g* for 2 h at 4°C.

Instead of resuspending the final pellet in culture medium, the pellet obtained after the 100,000 × *g* centrifugation was resuspended in 1 mL of PBS and used as input material for subsequent SEC using the Exo‐spin Midi Column Exosome Purification Kit (Protocol Version 7.7). Columns were equilibrated by the sequential addition of 2 × 10 mL of PBS and allowed to drain by gravity without centrifugation. The resuspended sample was then loaded onto the column, and elution was carried out following the protocol up to step D.13.

A total of 24 fractions were collected and subsequently pooled into three groups. Fractions 7–12, corresponding to the expected elution volume of EVs, were combined and used as the Col‐EV‐enriched fraction for downstream analyses. Fractions 1–6 (F1) were stored as well and used as flow‐through controls in downstream experiments.

### 4.4 Col‐EVs Characterization

To characterize Col‐EVs, the following techniques were used:

#### 4.4.1 Nanoparticle Tracking Analysis (NTA)

Isolated Col‐EVs were diluted 1:20 in sterile‐filtered PBS in order to reach the optimal concentration range for particle detection. Measurements were performed using an NTA system (NanoSight). All measurements were conducted at room temperature (25.0°C) under constant flow conditions with a flow rate of 40 µL/min. Measurement time was approximately 60 s per capture. A Blue488 laser and an sCMOS camera were used for all samples.

Acquisition and analysis parameters, including camera level, shutter speed, gain, frame rate (FPS), number of frames, and detection threshold, were individually adjusted for each sample to optimize particle visualization and tracking. The viscosity was set to that of water (0.9 cP). Data were analysed using the manufacturer's software (NTA 3.4.003), with automatic settings for blur size and maximum jump distance.

#### 4.4.2 Single‐Particle Interferometric Reflectance Imaging Sensor (SP‐IRIS)

In order to evaluate the expression of EV‐specific surface markers, we used the SP‐IRIS (ExoView) technology. For this, sample dilutions were prepared based on the concentration of particles/mL obtained previously through NTA, with the optimal concentration, as recommended by the manufacturer, being 10^9^ particles per chip for incubations. Each kit comprises eight chips (pre‐coated with capture antibodies or customizable), various solutions and detection antibodies anti‐tetraspanins, specifically anti‐CD9 (Alexa Fluor 488), anti‐CD81 (Alexa Fluor 594) and anti‐CD63 (Alexa Fluor 647).

For data analysis, fluorescent cut‐offs were modified from 200 a.u. (for red, far‐red and green channels) and from 400 a.u. (for blue channel) to limit the number of detected particles on MIgG to 50 events (for red, far‐red and green) or 100 events (for blue) following the technical instructions of the manufacturer using the ExoView Analyzer v3.2 (Unchained Laboratories).

#### 4.4.3 Transmission Electron Microscopy (TEM)

Samples were prepared on carbon‐coated copper grids for TEM analysis. Briefly, Col‐EV samples were diluted 1:10 in ultrapure water, followed by gentle vortexing to ensure homogeneity. Using fine forceps, a carbon‐coated copper grid was carefully placed on a Parafilm. Then, 10 µL of the diluted sample was applied onto the grid surface. Excess liquid was gently wicked away using filter paper to avoid damaging the grid or sample. The grids were then left to air dry at room temperature. To enhance contrast, grids were subsequently stained with one drop of 1% phosphotungstic acid solution for 1 min, followed by one drop of ultrapure water to wash off excess stain. The grids were then allowed to dry completely before imaging on a transmission electron microscope (model JEM2010) at the microscopy unit of the University of Santiago de Compostela (CACTUS).

### 4.5 Cell Viability Assays

Cell lines (LoVo, PANC1, CAFs, MDCK, HCT116 and HT29) were maintained in complete culture media under standard incubation conditions. LoVo cells were cultured in DMEM/F12 supplemented with 10% FBS and 5 mL of penicillin/streptomycin (P/S). PANC‐1 cells were maintained in high‐glucose DMEM with 10% FBS and 5 mL P/S. hTERT‐Immortalized primary rectal CAFs were cultured in DMEM Glutamax supplemented with 10% FBS, 1% insulin–transferrin–selenium (ITS), and 1% P/S. MDCK cells were maintained in DMEM Glutamax with 10% FBS and 5 mL P/S. HCT116 and HT29 cells were cultured in McCoy's 5A medium supplemented with 10% FBS and 5 mL P/S.

For viability assays, cells were seeded 1 day prior to treatment at densities of 10,000–30,000 cells per well in 96‐well plates or 50,000 cells per well in 24‐well plates, depending on the cell line. On Day 1, culture medium was removed and replaced with a mixture of Col‐EVs and fresh medium. For 96‐well plates, 50 µL of Col‐EVs suspension was diluted in 150 µL of medium; for 24‐well plates, 167 µL of Col‐EVs suspension was diluted in 500 µL of medium. The selected volumes correspond to final working concentrations of EVs (particles/µL) of 1.68 × 10^8^ for Col‐EVs, 2.22 × 10^8^ for Milk‐EVs, 1.72 × 10^8^ for SEC‐EVs and 7.39 × 10^6^ for F1 SEC‐EVs, measured by NTA. Cells were incubated with treatments for 96 h. Proliferation/viability was then assessed using alamarBlue cell viability reagent (ThermoFisher Scientific). Cells were incubated for 3 h with a 1:10 alamarBlue solution (diluted in complete medium), and then fluorescence was measured in a spectrophotometer using 580/590 nm (excitation/emission) filter settings.

Controls included untreated cells, cells treated with bovine serum albumin (BSA), the 70,000 × *g* centrifugation fraction (70k fraction), Col‐EVs isolated by SEC, and a control for the SEC procedure using the F1 fraction (pool of fractions 1–6). These flow‐through controls were included to assess the specificity of the effects of Col‐EVs.

### 4.6 Live‐Cell Imaging

Cells were seeded into 24‐well plates 1 day prior to imaging and incubated overnight at 37°C with 5% CO_2_. Time‐lapse live‐cell imaging was performed using a Celldiscoverer 7 automated microscope (Zeiss) equipped with a temperature‐ and CO_2_‐controlled incubation chamber (37°C, 5% CO_2_) to maintain physiological conditions throughout the experiment. Imaging was carried out using a Plan‐Apochromat 5×/0.35 objective lens. Brightfield images were acquired every 30 min over a 69‐h period, yielding a total of 138 time points per position. For each well, five distinct positions were imaged. Brightfield contrast was used with a light source intensity set at 10%.

### 4.7 EV Treatment and Flow Cytometry Analysis

LoVo and PANC1 cells were seeded at a density of 5 × 10^4^ cells per well in 24‐well plates 1 day prior to treatment. Cells were then treated with Col‐EVs for 96 h under standard incubation conditions (37°C, 5% CO_2_). After incubation, cells were harvested by trypsinization (0.05% trypsin‐EDTA, 5 min, 37°C), collected by centrifugation, and fixed in 4% paraformaldehyde (PFA) for 20 min at room temperature. Fixed cells were stained with Zombie NIR Fixable Viability Dye (BioLegend) at a 1:1000 dilution in PBS for 30 min at room temperature in the dark.

Flow cytometry acquisition was performed on a FACSCanto III cytometer (BD Biosciences) using BD FACSDiva Software Version 9.0. The APC laser was used for Zombie NIR detection, with voltage settings of 297 for APC, 187 for FSC‐A and 313 for SSC‐A. A total of 10,000 events were recorded per sample. Data were analysed using standard gating strategies with FlowJo software (v10.7.1, BD Life Sciences).

### 4.8 Proteomic Analysis

Proteomic analysis was carried out at the proteomics core facility of the Health Research Institute of Santiago de Compostela. For this, LoVo and PANC1 cells were seeded in 6‐well plates (10^5^ cells per well) and then treated for 48 h with either control, col‐EVs, and 70k fraction extracts. Treatments were conducted in triplicates.

Proteomic analyses were performed as previously described (Pereira‐Veiga et al. 2022; Mondelo‐Macía et al. 2024). Briefly, for EV proteomics, 15 µg of Col‐EVs or 70k fraction proteins were mixed with a lysis buffer in ratio 1:1 boiled and concentrated in a resolving 10% SDS‐PAGE gel (Abramowicz et al. 2018). For cell line proteomics, after treatment, cells were lysed, protein quantified and preconcentrated in an SDS‐PAGE gel, followed by protein band staining (Sypro Ruby fluorescent staining; Lonza, Basel, Switzerland) and band excision. Gel pieces were reduced (10 mM DTT; Sigma–Aldrich, St. Louis, MO, USA) and alkylated (55 mM iodoacetamide; Sigma–Aldrich, St. Louis, MO, USA). Then, we performed in‐gel tryptic digestion, as previously described (Pereira‐Veiga et al. 2022; Mondelo‐Macía et al. 2024). Peptides were extracted [50% ACN/0.1% TFA (×3) and ACN (×1)], pooled, concentrated in a SpeedVac and stored at −20°C.

#### 4.8.1 Mass Spectrometric Analysis (DDA Acquisition)

DDA analysis was made as previously described (Pereira‐Veiga et al. 2022). Briefly, digested peptides (over 4 µg of each sample: Col‐EVs, 70K fraction, LoVo, PANC1) were separated using Reverse Phase Chromatography. A 90 min gradient ranging from 2% to 90% mobile phase B, was created using a micro liquid chromatography system (Eksigent Technologies nanoLC 400, Sciex) coupled to a high‐speed Triple TOF 6600 mass spectrometer (Sciex) with a micro flow source. Data acquisition was performed by a TripleTOF 6600 System (Sciex, Foster City, CA) using a data‐dependent analysis (DDA) workflow. Source and interface conditions were the following: ionspray voltage floating (ISVF) 5500 V, curtain gas (CUR) 25, collision energy (CE), 10 and ion source gas 1 (GS1) 25. The instrument was operated with Analyst TF 1.7.1 software (Sciex, USA). Switching criteria was set to ions greater than mass‐to‐charge ratio (m/z) 350 and smaller than m/z 1400 with charge state of 2–5, mass tolerance of 250 ppm and an abundance threshold of more than 200 counts per second (cps). Previous target precursor ions were excluded for 15 s. The instrument was automatically calibrated every 4 h using tryptic peptides from PepCalMix as an external calibrant.

After MS/MS analysis (MS2 data), data files were processed using ProteinPilot 5.0.1 software from Sciex, which uses the algorithm Paragon for database search and Progroup for data grouping. Data were searched using a Bovine specific Uniprot database (https://www.uniprot.org/) specifying iodoacetamide at cysteine alkylation as variable modification and methionine oxidation as fixed modification. FDR was performed using a non‐lineal fitting method, displaying only those results that reported a 1% Global FDR or better (Shilov et al. 2007).

#### 4.8.2 Protein Quantification by SWATH (Sequential Window Acquisition of All Theoretical Mass Spectra)

For label‐free quantitative proteomics, we performed an MS analysis by sequential window acquisition of all theoretical mass spectra (SWATH‐MS), as previously described (Pereira‐Veiga et al. 2022; Mondelo‐Macía et al. 2024). First, a unique peptide pool was created by mixing equal amounts of peptides from each sample type (LoVo, PANC1). This pool was analysed by LC‐MS/MS on a TripleTOF 6600 LC‐MS/MS system via a data‐dependent acquisition (DDA) method in order to create a SWATH‐MS spectral library. Only proteins and peptides with <1% FDR were included in this library (Shilov et al. 2007). Then, 4 µg of peptides derived from the individual samples were analysed by SWATH‐MS method. SWATH‐MS acquisition was performed on a TripleTOF 6600 LC‐MS/MS system via a data‐independent acquisition (DIA) method. The whole 400 to 1250 m/z range was covered in 100 steps with spectral windows of variable width (1 m/z overlap). Peak extraction was carried out with PeakView software (version 2.2; Sciex, Redwood City, CA, USA) and scored using the PeakView SWATH Acquistion MicroApp (version 2.0; Sciex, Redwood City, CA, USA). The integrated peak areas were exported to the MarkerView software (version 1.3, Sciex, Redwood City, CA, USA). To ensure a more accurate comparison between samples, well‐known endogenous peptides were used during data alignment to compensate for small variations in both mass and retention times. The amount of each protein in every sample was calculated as the averaged area sums of 10 peptides per protein and 7 transitions per peptide. Then, an averaged MS peak area of each protein was calculated. Data normalisation was carried out with the most likely ratio normalisation (MLR) method. As part of the initial analysis, the MarkerView software (version 1.3, Sciex, Redwood City, CA, USA) also allowed a PCA to see how well each protein distinguishes between groups. We considered the proteins differentially expressed with a *p* value *<*0.01 and a fold change of 1.3/−1.3.

The mass spectrometry proteomics data have been deposited to the ProteomeXchange Consortium via the PRIDE (Perez‐Riverol et al. 2025) partner repository with the dataset identifier PXD066002 for Col_EVs and 70k fraction, and  PXD066009 for treated cell lines.

Functional analyses were performed using the FunRich open access software (Functional Enrichment analysis tool) for functional enrichment and interaction network analysis (http://funrich.org/index.html) (Fonseka et al. 2021), and Gene Ontology analyses were performed using Genecodis4 (https://genecodis.genyo.es/) (Garcia‐Moreno et al. 2022).

Volcano plots were generated using VolcaNoseR (Tang et al. 2023), and bubble plots were built using SRplot (Tang et al. 2023).

### 4.9 Validation of Proteomic Candidates by Qpcr

LoVo cells were cultured in 6‐well plates and treated with ColEVs for 96 h, maintaining the same ColEV concentration used in proteomic experiments. After the treatment period, cells from each condition (Control and ColEV‐treated) were scraped separately and centrifuged to obtain cell pellets, which were frozen until further processing. Total RNA was extracted using the QIAwave RNA Mini Kit (Qiagen), following the manufacturer's instructions. The concentration and purity of the isolated RNA were determined using a NanoDrop spectrophotometer.

A total of 100 ng of RNA from each sample was loaded into a 96‐well PCR plate together with TaqMan reagents and the corresponding probes for the selected target genes. Each reaction was prepared in a final volume of 20 µL, consisting of TaqMan Fast Virus 1‐Step Master Mix, probe, sample, and nuclease‐free water, according to the manufacturer's recommendations and run in a QuantStudio 3 Real‐Time PCR System (Applied Biosystems). Relative gene expression was normalized to *B2M*n for each probe.

### 4.10 Establishment of PDAC Organoid Cultures From Fine Needle Aspirates

PDAC tumour tissue was obtained through endoscopic ultrasound guided fine‐needle biopsy (EUS‐FNB) from patients after signing the informed consent previously approved by the “Comité de Ética de Galicia”, under the approval number 2023‐016. A team of gastroenterologists and endoscopists explained the project and procedures to the patient before their acceptance to participate in the study. Sample was initially collected in DMEM‐F12 (Sigma–Aldrich) supplemented with 0.1% BSA (Thermo Scientific), in a 50 mL falcon tube. Tissue was then centrifugated at 200 RCF for 5 min at 4°C and washed once. After that RBC lysis buffer (Thermo Scientific) was added and incubated at 37°C for 10 min. Once the blood cells surrounding the tumoral tissue were eliminated, sample was washed again by centrifugation. Eventually, the tissue filament was transferred to a petri dish, where it was minced into small pieces of approximately 1 mm^3^ with a scalpel. The obtained fragments were washed once and then embedded in GFR Matrigel (Corning) for seeding in domes of 50 µL in 24 well culture plates prewarmed for 30 min at 37°C in the incubator. These domes were kept at 37°C for 20 min allowing Matrigel to polymerize and then 500 µL of culture medium (DMEM/F12 medium (Merck Millipore), supplemented with Glutamax (1:100, Thermo Scientific) antibiotic/antimycotic (1:100, Thermo Scientific), B27 (1:50, Thermo Scientific), N‐acetyl‐L‐cysteine (1.25 mM, Sigma), Wnt3a (100 ng mL^−1^, MedChemExpress), RSPO1 (100 ng mL^−1^, Peprotech), Noggin (100 ng mL^−1^, Peprotech), epidermal growth factor (EGF, 50 ng/mL, Peprotech), Gastrin (10 nM, Sigma), fibroblast growth factor 10 (FGF10, 100 ng/mL), Nicotinamide (10 mM, Sigma), A83‐01 (0.5 µM, Sigma) and Y‐27632 (10 µM, MedChemExpress) was added to each well.

### 4.11 Immunofluorescence Staining and Imaging

LoVo and PANC1 cells were seeded at a density of 5 × 10^4^ cells per well in 24‐well plates 1 day prior to treatment. Cells were then treated with Col‐EVs for 96 h under standard conditions (37°C, 5% CO_2_). BSA was used as a vehicle control. Following treatment, cells were fixed with 4% PFA for 20 min at room temperature and subsequently washed with PBS.

An additional recovery condition was included in which cells were treated with Col‐EVs for 96 h, followed by a 48‐h incubation period in complete medium (medium change performed to remove Col‐EVs), and subsequently processed for staining as described.

For other immunofluorescence studies on different cell lines, including repeated experiments with LoVo and PANC1, as well as CAF/CRC‐hTERT, MDCK, HCT116 and HT29 cells, 8‐well chamber slides (Ibidi) were used. Cells were seeded 1 day prior to treatment at densities of 25,000–30,000 cells per well, depending on the cell line. Col‐EVs were added (100 µL), maintaining a total volume at 300 µL per well. LoVo cells were incubated for up to 96 h, while all other cell lines were incubated for 24 h, as the treatment had reached its observable effect by this time. Following treatment, cells were fixed with 4% PFA for 20 min at room temperature and subsequently washed with PBS.

Immunofluorescence analysis was performed on organoids grown for 3 days in Matrigel on 8‐well chamber slides (Ibidi) and incubated with the desired EVs treatments for 96 h. Eventually, domes were washed with PBS (Sigma) and fixed in 2.5% PFA/0.1% glutaraldehyde (ThermoFisher) for 1 h and subsequently washed three times with PBS.

#### 4.11.1 Staining

Fixed cells were permeabilized with 0.5% Triton X‐100 (Sigma) for 1 h and subsequently blocked with PBS, 3% BSA for 1 h. Then they were stained with DAPI (1:200) (ThermoFisher), phalloidin‐CruzFluor 633 (1:500) (Santa Cruz Biotechnology Ref. sc363796), and Ki‐67 (D3B5) Rabbit mAb (Alexa Fluor 488 Conjugate) (1:200) (Cell Signaling Technology Ref. #11882) for 1 h at room temperature in the dark, followed by three washes with PBS.

Organoids were permeabilized with PBS, 1% Triton X‐100 (Sigma) for 1 h and subsequently blocked with PBS, 3% BSA for 1 h. Then they were stained with DAPI (1:500) (ThermoFisher), phalloidin‐CruzFluor 633 (1:500) (Santa Cruz Biotechnology Ref. sc363796) and Ki‐67 Monoclonal Antibody (SolA15), Alexa Fluor 488, eBioscience (1:100), left overnight 4°C in the dark, followed by three washes with PBS.

#### 4.11.2 Confocal Imaging

Stained conditions were imaged using an inverted confocal microscope (LEICA SP8) using laser lines 405, 488, 552 and 633 nm and the following objectives: HC PL APO CS2 10×/0.40, HC PL APO CS2 20×/0.75, HC PL APO CS2 40x/1.30 OIL and HC PL APO CS2 63×/1.4 OIL. Images were processed using ImageJ (v2.14.0/1.54f).

### 4.12 Image Analysis

Image analysis was performed using Fiji/ImageJ (v2.14.0/1.54f). Brightfield images were converted to 8‐bit format, and contrast was enhanced using the “Enhance Contrast” function with 0.35% saturated pixels and the Normalize option enabled, applied uniformly across all images. In cases where Z‐stacks were acquired, maximum intensity Z‐projections were generated. The background was subtracted using a rolling ball radius of 35 pixels. Binary masks were generated using the “Make Binary” function with Otsu thresholding, assuming a dark background. Particle analysis was conducted using the “Analyze Particles” tool, and the following parameters were extracted and summarized for each image: count, area, average size, percent area, mean intensity, mode, perimeter and integrated density (IntDen). For quantification of Ki67 relative to nuclei, cells were counted manually using the Cell Counter tool with DAPI and Ki67 channels, and in cases where Z‐stacks were acquired, a representative Z‐stack was selected.

### 4.13 Osmotic Shock Assay

Osmotic solutions were prepared by diluting D‐sucrose (Fisher Scientific) in cell culture medium to final concentrations of 0.4, 0.5 and 0.6 M. All solutions were sterilized by filtration through a 0.22 µm syringe filter prior to use. Osmolarity was measured using an OsmoSpecial1 osmometer.

To test osmotic stress‐induced chromatin condensation, LoVo cells were seeded at a density of 25,000–30,000 cells per well in 8‐chamber ibidi slides in a final volume of 300 µL 1 day prior to treatment. Cells were then exposed to D‐sucrose for 1 h. Following treatment, cells were fixed with 4% PFA for 20 min at room temperature and subsequently washed, permeabilized and processed for immunofluorescence analysis.

### 4.14 Comparative Drug Response Assay

PANC1 cells were seeded in 96‐well plates at a density of 10,000 cells per well and allowed to adhere overnight. Cells were then treated under co‐treatment conditions for 96 h, with all conditions performed in triplicate.

The following drug concentrations were tested:

- Actinomycin D: 1 ng/mL and 100 ng/mL
- 5‐Fluorouracil (5‐FU): 1.5 and 15 mM

After 96 h of treatment, cell proliferation and viability were assessed using the alamarBlue Cell Viability Reagent (Thermo Fisher Scientific). Cells were incubated with a 1:10 dilution of alamarBlue in complete medium for 3 h. Fluorescence was then measured using a microplate reader at 580 nm excitation and 590 nm emission.

### 4.15 Rescue Assay Using Tazemetostat

For the rescue assays, PANC1 cells were seeded in 8‐well chamber Ibidi slides 1 day prior to treatment. Organoids were previously established in 8‐well Ibidi chamber slides. Cells and organoids were treated with Tazemetostat (40 µM) in culture medium. This dose was selected based on a dose–response viability curve in PANC1 and LoVo. Both cells and organoids were initially treated with Tazemetostat alone for 24 h. Subsequently, while maintaining the drug in the medium, Col‐EVs were added (100 µL) to reach a final volume of 300 µL.

PANC‐1 cells were co‐treated with Col‐EVs for an additional 24 h and then fixed and stained for imaging analysis. Organoids were treated for a total of 96 h, after which they were fixed, permeabilized, and analysed by immunofluorescence.

The following controls were included: BSA, Col‐EVs alone (without Tazemetostat), and Tazemetostat alone.

### 4.16 Data Analysis

Data visualization and statistical analyses were performed using GraphPad Prism 10. Statistical tests were selected based on data distribution. Experimental designs as well as the number of biological and technical replicates are specified in the corresponding figure legends. A *p* value <0.05 was considered statistically significant.

## Author Contributions

**Bruno Puga**: investigation, methodology. **Yessica Domínguez‐Novoa**: resources. **Julio Iglesias‐García**: resources. **Brígida Hermida**: conceptualization, resources. **Rafael López‐López**: conceptualization, resources, writing – review and editing. **Claudia Betsabé Huesa‐Carballo**: investigation, methodology, validation, formal analysis. **Susana Bravo**: methodology, software, formal analysis. **Manuel Rodríguez‐Pérez**: methodology, investigation. **Isolina Raña**: resources, conceptualization. **María Teresa Antelo**: conceptualization, resources. **Miguel Abal**: writing – review and editing, conceptualization, investigation, resources, supervision. **Jorge Barbazán**: conceptualization, investigation, funding acquisition, writing – original draft, writing – review and editing, visualization, formal analysis, project administration, data curation, supervision, resources

## Conflicts of Interest

The authors declare no conflicts of interest.

## Supporting information




**Supporting Information**: jev270361‐sup‐0001‐VideoS1.avi

**Supporting Information**: jev270361‐sup‐0002‐SuppMat.xlsx

**Supporting Information**: jev270361‐sup‐0003‐FigureS1‐S8.docx

## Data Availability

The data that support the findings of this study are openly available in ProteomeXchange Consortium via the PRIDE partner repository at https://proteomecentral.proteomexchange.org/ui, reference number PXD066002 and PXD066009.
