# Supplementary material for: Bovine Colostrum‐Derived Extracellular Vesicles Impair Cancer Cell Proliferation Through Transcriptional Dysregulation
Source: J Extracell Vesicles. 2026 Sep 1;15(9):e70361. doi: 10.1002/jev2.70361 (PMC13533780; doi:10.1002/jev2.70361)
Supplement: Supplementary file 1 [file JEV2-15-e70361-s001.docx]

**Supplementary Fig. 1**. Schematic representation of the protocol for isolation of Col-EVs by ultracentrifugation.

**Supplementary Fig. 2.** Representative images from transmission electron microscopy (TEM) showing the contents of both ultracentrifugation fractions (A) Col-EV 100k fraction (B) 70k fraction. (C) Violin plots of the quantification of particle size and concentration (N=4), respectively. (D) Quantification of particle concentration using SP-IRIS (N=1). MIgG1 indicates the signal obtained using an isotype control. Error bars: SD. (E) Co-expression analysis showing heterogeneity in tetraspanin profiles across the Col-EV population (N=1).

**Supplementary Fig. 3:** (A) Representative confocal images of F-actin (magenta) and DAPI-stained (cyan) LoVo cells demonstrating vertical multilayering and reduced spreading upon Col-EV treatment. Scale bar: 50 µm for main images and 20 µm for orthogonal sections. (B) Cell death analysis of PANC1 and LoVo cells treated or not with Col-EVs. Error bars: SD. (N=2). (C) Representative confocal images of DAPI-stained nuclei from different cells, control and Col-EV treated. Scale bar: 5 µm. (D) Quantification of the standard deviation of the nuclear signal/nucleus. Data represents at least 12 analyzed nuclei from each cell line. N=2 independent experiments. **p<0.01; ANOVA test/Sidak's multiple comparisons test; HT29 95% CI [-9.872,-1.389].

**Supplementary Fig. 4. Characterization of Col-EVs isolated by SEC and their functional effects.** (A,B) Protocol for the isolation of Col-EVs using Size Exclusion Chromatography (B) following sample preparation by ultracentrifugation and EDTA-based casein clearing (A). (C) NTA characterization showing similar particle size (left panel) but lower EV concentration (right panel) (*p<0.05) when comparing ultracentrifugation alone with SEC isolation, ANOVA Kruskal-Wallis test/Dunn’s multiple comparisons. (D) Confocal images confirming Col-EV effects independent of isolation method; the initial 1-6 SEC fractions (SEC F1) were used as flow-through controls. Scale bars: 100µm (E) Quantification of Ki67-positive cells as a fraction of total DAPI-positive cells shows no significant differences between the two isolation methods. **p<0.01; ***p<0.001; ANOVA Kruskal-Wallis test/Dunn’s multiple comparisons. Data from at least 11 images from N=2 independent experiments. (F) Representative confocal images of DAPI-stained nuclei showing the chromatin condensation effect induced by Col-EVs across different isolation methods. Scale bars: 5µm. (G) Quantification of the % nuclear area covered by DNA (left panel), as well as the standard deviation of the nuclear signal/nucleus (right panel). Data represents at least 11 analyzed nuclei from N=2 independent experiments. **p<0.01, ***p<0.001; ANOVA Kruskal-Wallis test/Dunn’s multiple comparisons.

**Supplementary Fig. 5: Comparison of Col-EVs and paired Milk-EVs effects** (A) Left panel: NTA characterization of Milk-EVs, and paired Col-EVs isolated by ultracentrifugation and SEC. Right panel: Concentration of Milk-EVs, and Col-EV from ultracentrifugation and SEC. (*p < 0.05, ANOVA Kruskal-Wallis test/Dunn’s multiple comparisons). (B) Representative phase-contrast and confocal microscopy images showing an intermediate effect induced by Milk-EVs compared with paired Col-EVs. Scale bars: 100 µm (C) Quantification of Ki67-positive cells as a fraction of total DAPI-positive cells (***p<0.001; N at least 2 for control and Col-EVs, N = 1 for Milk-EVs; ANOVA Kruskal-Wallis test/Dunn’s multiple comparisons). (D) qPCR validation analyses of selected candidates from proteomic analyses. (E) Representative confocal images of DAPI-stained nuclei showing the chromatin condensation phenotype in Col-EVs and paired Milk-EVs treated cells. Scale bars: 5 µm. (F) Quantification of the nuclear signal standard deviation per nucleus in Col-EV and Milk-EV-treated cells (*p < 0.05; N at least 2 for control and Col-EVs, N = 1 for Milk-EVs; ANOVA Kruskal-Wallis test/Dunn’s multiple comparisons).

**Supplementary Fig. 6. Effect of Col-EVs in cells entering mitosis.** (A) Representative confocal images of mitotic figures in LoVo cells, stained for DNA (DAPI, blue), Ki67 (green) and F-Actin (Phalloidin, magenta), from control, Col-EV- treated or recovered conditions. Scale bar: 10 µm (B) Representative line-scan analysis of DAPI signal intensity in metaphasic chromosomal plates from images in panel. (C) Quantification of the standard deviation of DAPI intensity across line scans in metaphase plates, where lower standard deviation in Col-EV-treated cells indicates reduced peak variability consistent with hypercompaction and loss of individualized chromosome structure. ***p<0.001, *p<0.05, Kruskal-Wallis ANOVA test/Dunn’s multiple comparisons. (D) Transversal intensity profiles of DAPI and Ki67 across nuclei in control, Col-EVs, and recovered in LoVo cells, indicating aberrant mitotic distribution.

**Supplementary Fig. 7. Osmotic stress assay.** (A) Osmolarity quantification of D-sucrose increasing concentration samples used for osmotic shock induction (N = 2). (B) Representative confocal images of DAPI-stained nuclei from LoVo cells after 1 h of osmotic shock induction. Scale bars: 5 µm. (C) Osmolarity measurement of controls and different EV solutions.

.

**Supplementary Fig. 8. Modeling PDAC Organoid Response to Col-EVs and Rescue with Tazemetostat.** Representative tile-scans of Matrigel drops containing organoids treated with the indicated conditions. Right panels, insets os selected regions. Scale bars: 1 mm.
